# Closed form solution of nonlinear oscillation of a cantilever beam using *λ*-symmetry linearization criteria

**DOI:** 10.1016/j.heliyon.2022.e11673

**Published:** 2022-11-16

**Authors:** Raed Ali Mara'Beh, Ahmad Y. Al-Dweik, B.S. Yilbas, M. Sunar

**Affiliations:** a Department of Mathematics, Foundation Program, General Studies, Qatar University, Doha, 2713, Qatar; b Department of Mathematics, Statistics and Physics, College of Arts and science, Qatar University, Doha, 2713, Qatar; c Mechanical Engineering Department, King Fahd University of Petroleum and Minerals, Dhahran, 31261, Saudi Arabia; d Ankara Yildirim Beyazit Universitesi, Turkey

**Keywords:** Cantilever beam, Non-linear oscillation, Closed form solution, Lie-Tresse linearization

## Abstract

A mechanical system, in general, undergoes vibrational motion when the system is subjected to a tension or an external force. One of the examples of such a system is a cantilever beam when it is exposed to a bending action. When the tension is released, the cantilever beam suffers from the oscillations until the strain energy is totally released through the damping characteristics of the cantilever beam. Depending on the stiffness and damping factors of the beam, the vibrational motion can be non-linear; in which case, the analytical solution becomes challenging formulating the flexural characteristics of the beam. Although numerical solution for the non-linear problem is possible, the analytical solution provides useful information between the mechanical response and the cantilever beam characteristics. In the present study, the analytical solution of the non-linear equations governing the motion of the cantilever beam is presented. The governing equation is linearized incorporating the Lie-Tresse linearization method. The closed form solution for the displacement of the cantilever beam is reduced to a linear solution after introducing the appropriate beam characteristics. The dynamic behavior of the flexural motion due to non-linear and linear cantilever beams are compared.

## 1 Introduction

Cantilever beams are widely used in mechanical systems [1] and building constructions [2] because of their unique flexural characteristics. Depending on the beam material properties and its structural homogeneity, damping and stiffness characteristics of the cantilever beam change. In some cases, variable damping and stiffness parameters of the beam are designed to damp and release the mechanical energy within the desired time frame. This arrangement requires fabricating a non-linear cantilever beam with varying damping coefficient and stiffness. In general, the cantilever beam is anchored at one end and carries the load to the support where it is forced against by moment and shear stress. The non-linear properties of the beam results in the flexural motion of the beam with high damping rate when the load is released from the edge, which is under tension. Although a numerical solution of the flexural characteristics of the cantilever beam with a non-linear damping and stiffness is possible, the closed form solution to the problem offers several advantages. Firstly, the closed form solution provides the functional relation between the non-linear properties of the beam and the flexural characteristics. Secondly, it reduces the computational efforts required for the solution. Since the governing equation of motion describing the flexural performance of the non-linear cantilever beam is in a non-linear form, the closed form elucidation of the problem becomes challenging for the general solution of the problem. Consequently, study into analytical solution of the flexural characteristics of non-linear cantilever beam becomes essential. Considerable research studies were carried out to examine analytically solution for the flexural characteristics of the non-linear cantilever beams. The location of a grazing in the constrained motion of a nonlinear cantilever beam was studied by Dick et al. [3]. They presented the non-linear phenomenon by using phase portraits, Poincare' sections, and spectral analysis. They indicated that for off-resonance excitation at two and a half times the fundamental frequency, the response of the oscillating cantilever experienced a period doubling as the separation distance or clearance between the beam axis and the contact surface was decreased. Non-linear parametric amplification and attenuation in a base-excited cantilever beam were investigated by Kumar et al. [4]. They demonstrated that with the proper selection of various system parameters, both vibration amplification and attenuation could be efficiently achieved. Nonlinear normal modes of a parametrically excited cantilever beam were examined by Yabuno and Nayfeh [5]. They showed that the system had nonlinear modes, as defined by Rosenberg, even in the presence of the parametric excitation. In addition, they determined the spatial correction to the linear mode shape due to the effects of the inertia and curvature nonlinearities and the parametric excitation. Non-linear vibration of a magneto-elastic cantilever beam with tip mass was studied by Pratiher et al. [6]. They used the method of multiple scales to determine the instability region and frequency response curves of the system. Higher-harmonic effects of a slender vertical cantilever beam to fully nonlinear regular wave forcing was investigated by Bredmose et al. [7]. They conducted the parametric studies of the response dependence to relative forcing period and demonstrated that for waves with a maximum height of 85%, the third-harmonic response increased significantly when the depth was decreased from deep depth conditions into moderate depth. Nonlinear vibration control of a cantilever beam by a nonlinear energy sink was examined by Ahmadabadi and Khadem [8]. Their findings revealed that the realization of nonlinear vibration control through one-way irreversible nonlinear energy pumping and optimizing the system parameters resulted in acquiring up to 89% dissipation of the ungrounded system energy imposed by a shock excitation. Some approximate solutions for the large deflection nonlinear problem of a cantilever beam subjected to a terminal follower force and non-linear pendulum model were presented by Vazquez-Leal et al. [9]. They used nonlinearities distribution homotopy perturbation approach and combinations with Laplace-Pade' posttreatment to provide some approximate solutions for both nonlinear models. They showed that the high accuracy of the proposed cantilever solutions were consistent with the other reported solutions. The solutions to nonlinear vibration of cantilever beam via homotopy perturbation method were presented by Ma et al. [10]. The comparison of the result obtained by the homotopy perturbation method with numerical solutions revealed that only the first order approximation leads to a higher accurate solution. Non-linear analysis of a self-excited cantilever beam was carried out by Kaneko et al. [11]. They designed a van der Pol type self-excited cantilever beam by applying the non-linear feedback proportional to the squared deflection and the velocity while incorporating the steady state response of the cantilever beam. Non-linear behavior analysis of micro cantilever beam subject to electrostatic loading was realized by Liu et al. [12]. They showed that the hybrid differential transform approach provides an accurate and efficient computational analysis to the complex non-linear performance of both the current micro cantilever beam system and other micro-scale electrostatically-actuated structures.

Analysis of the nonlinear response of a cantilever beam under deterministic and random excitation was presented by Benedettini et al. [13]. They derived the differential equations of phase and amplitude and applied a linearization technique to evaluate the second order statistics. The findings were validated through digital simulations on a Duffing-Rayleigh oscillator incorporating the cantilever beam with tip force. Vibration characteristics of a flexible cantilever beam and limitations of an equivalent linearized method were investigated by Li et al. [14]. They considered the large deformation and developed an equivalent linearization method to calculate the vibrating response of the beam. They showed that the changes of measured values of the frequency response function were very small when the ratio of tip dynamic displacement amplitude to static deformation amplitude was less than 10%. Non-linear normal modes of a continuous cantilever beam with non-linear energy sink absorber were examined by Yong and Yi [15]. They used Galerkin's and Rausher's methods to obtain non-linear normal modes analytically and, from the comparison of analytical and numerical results, they indicated that the nonlinear normal modes were present. Although non-linear behavior of the damping system in relation to nonlinear energy sink was investigated previously [8], the main focus was the behavior of cantilever beam with the external source excitations and various conditions due to the tension of the free end were left obscure. In addition, numerical studies for the cantilever beam flexural characteristics were carried out under different loading characteristics [16], [17], [18]. However, the studies were limited to the practical applications and the fundamental solution for the non-linear motion was not included. Therefore, in the present study, the closed form solution for the non-linear equation of motion governing the cantilever beam displacement is presented. Lie-Tresse linearization method was used to linearize the governing equation of motion. In [21], a new *λ*-symmetry linearization criteria was established for second-order differential equations. In recent years, much attention has been done to the *λ*-symmetry linearization for solving nonlinear equations [22], [23]. The analytical study is extended to include three conditions at which the cantilever beam is subjected to prior to the flexural motion. These are: (i) initial displacement and the velocity for some non-zero instant are known, (ii) initial displacement and the non-zero displacement for some non-zero instant are known, and (iii) initial displacement and initial velocity are known. The analytical solution obtained for the cantilever beam displacement is reduced to a linear solution after introducing the appropriate beam damping and stiffness parameters. The findings of reduced form are compared with those presented in the open literature for the linear motion of the beam.

## 2 Mathematical analysis

**Figure 1:**
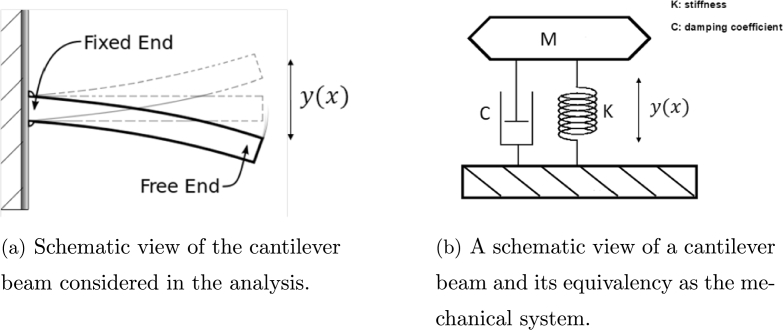
A schematic view of a cantilever beam and its equivalency as the mechanical system.

The cantilever beam with non-linear damping and stiffness parameters undergoes a non-linear flexural motion when the tension at the free end is released. Since the cantilever beam under consideration has considerably smaller thickness than its length, the beam can be considered to be a thin plate and the non-linear cantilever flexural motion problem reduces to one-dimensional form. This situation can also be represented as a mass supported by non-linear damper and stiffness as shown in Figs. 1(a) and 1(b).

Since the governing equation of the motion is non-linear, a linearization scheme needs to be introduced for the solution of the problem. For the linearization problem of second order ordinary differential equation (ODE)'s via point transformations, Lie [19] showed that any second-order ODE

(1) $$y^{\prime \prime }=f(x,y,y^{\prime })$$

obtainable from the free particle equation

$$u_{tt}=0$$

by change of variables

(2) $$t=\phi \left(x,y\right),\;\;\;u=\psi \left(x,y\right),\;\;\;J=\phi_{x}\psi_{y}-\phi_{y}\psi_{x}\neq 0,$$

should be at most cubic in the first derivative, i.e. it has the form

(3) $$y^{\prime \prime }+F_{3}(x,y)y^{\prime \;3}+F_{2}(x,y)y^{\prime \;2}+F_{1}(x,y)y^{\prime }+F(x,y)=0,$$

with the coefficients $F(x,y),\;F_{1}(x,y),\;F_{2}(x,y)$ and $F_{3}(x,y)$ satisfying the following system of partial differential equations

$$F_{3}(x,y)=A,\;F_{2}(x,y)=B+2w,\;F_{1}(x,y)=P+2z,\;F(x,y)=Q,$$

where

$$\begin{array}{ccc} A=\frac{\phi_{y}\psi_{yy}-\psi_{y}\phi_{yy}}{\phi_{x}\psi_{y}-\phi_{y}\psi_{x}},\; & B=\frac{\phi_{x}\psi_{yy}-\psi_{x}\phi_{yy}}{\phi_{x}\psi_{y}-\phi_{y}\psi_{x}},\; & w=\frac{\phi_{y}\psi_{xy}-\psi_{y}\phi_{xy}}{\phi_{x}\psi_{y}-\phi_{y}\psi_{x}},\\ Q=\frac{\phi_{x}\psi_{xx}-\psi_{x}\phi_{xx}}{\phi_{x}\psi_{y}-\phi_{y}\psi_{x}},\; & z=\frac{\phi_{x}\psi_{xy}-\psi_{x}\phi_{xy}}{\phi_{x}\psi_{y}-\phi_{y}\psi_{x}},\; & P=\frac{\phi_{y}\psi_{xx}-\psi_{y}\phi_{xx}}{\phi_{x}\psi_{y}-\phi_{y}\psi_{x}}. \end{array}$$

Lie [19] also found the following over-determined system of four equations

(4) $$\begin{array}{c} w_{x}=zw-FF_{3}-\frac{1}{3}\frac{\partial F_{1}}{\partial y}+\frac{2}{3}\frac{\partial F_{2}}{\partial x},\\ w_{y}=-w^{2}+F_{2}w+F_{3}z+\frac{\partial F_{3}}{\partial x}-F_{1}F_{3},\\ z_{x}=z^{2}-F_{1}z-Fw+\frac{\partial F}{\partial y}+FF_{2},\\ z_{y}=-zw+FF_{3}-\frac{1}{3}\frac{\partial F_{2}}{\partial x}+\frac{2}{3}\frac{\partial F_{1}}{\partial y}, \end{array}$$

which are called the Lie conditions. The compatibility of Lie's conditions gives the following well known Lie-Tressé linearization test for the ODEs of the form (3), viz.

(5) $$\begin{array}{c} \frac{\partial^{2}F_{1}}{\partial y^{2}}-2\frac{\partial^{2}F_{2}}{\partial y\partial x}+3\frac{\partial^{2}F_{3}}{\partial x^{2}}-3\frac{\partial F_{1}}{\partial x}F_{3}-3F_{1}\frac{\partial F_{3}}{\partial x}+6\frac{\partial F}{\partial y}F_{3}+3F\frac{\partial F_{3}}{\partial y}-F_{2}\frac{\partial F_{1}}{\partial y}\\ \;+2F_{2}\frac{\partial F_{2}}{\partial x}=0\\ \frac{\partial^{2}F_{2}}{\partial x^{2}}-2\frac{\partial^{2}F_{1}}{\partial y\partial x}+3\frac{\partial^{2}F}{\partial y^{2}}+3\frac{\partial F}{\partial y}F_{2}+3F\frac{\partial F_{2}}{\partial y}-3\frac{\partial F}{\partial x}F_{3}-6F\frac{\partial F_{3}}{\partial x}+F_{1}\frac{\partial F_{2}}{\partial x}\\ \;-2F_{1}\frac{\partial F_{1}}{\partial y}=0. \end{array}$$

It was Tressé [20] who first obtained the invariant criteria (5).

In [21], new *λ*-symmetry linearization criteria for second order ODEs have been provided as follows

Theorem *A scalar second-order ODE* (1) *is linearizable via point transformations* (2) *if and only if it has the cubic in first derivative form* (3) *with the λ-symmetries equivalent to the canonical pair* $\left(\frac{\partial }{\partial y},\lambda_{1}\right)$ *for* $\lambda_{1}=-F_{3}\;y^{\prime \;2}-\left(F_{2}-w\right)y^{\prime }-z$ *and the transformations ϕ and ψ satisfying the system*

(6) $$\begin{array}{c} S_{yy}+\left(2w-F_{2}\right)S_{y}+F_{3}S_{x}=0\\ S_{xy}+w\;S_{x}-z\;S_{y}=0\\ S_{xx}+\left(F_{1}-2z\right)S_{x}-F\;S_{y}=0 \end{array}$$

*where w and z are auxiliary functions.*

In general, the approach of obtaining the general local linearization transformations for nonlinear ODE of the form (3) is given as follows [21]:

1. Check if the coefficients of the ODE (3) satisfy the Lie linearization test (5).
2. Find any particular solution for w(x,y) and z(x,y) of the system (4).
3. Find the values of *ϕ* and *ψ* satisfy the system (6) for the evaluated w(x,y) and z(x,y).
4. Since the free particle equation $u_{tt}=0$ has the general solution $u(t)=s_{1}t+s_{2}$, then the local transformations (2) lead to the following general solution of the ODE (3)
  $$\psi (x,y)=s_{1}\phi (x,y)+s_{2}.$$

## 3 Analysis

The nonlinear ODE

(7) $$m\;y^{\prime \prime }+c\;y^{\prime }+k\;y=0$$

where $c=c_{0}+c_{1}y$, and $k=k_{1}+k_{2}y+k_{3}y^{2}$, satisfies the Lie linearization test (5) if and only if $k_{2}=\frac{c_{0}\;c_{1}}{3m}$, and $k_{3}=\frac{c_{1}^{2}}{9m}$. It should be noted that in the case of metallic materials, the damping coefficient and stiffness can be simplified through a polynomial form of displacement. In this case, the following are considered for simplicity to resemble flexural properties of a cantilever beam. Hence, we will approximate the values of $k_{2}$ and $k_{3}$ to make the ODE (7) linearizable, so that it can be written as

$$y^{\prime \prime }+\frac{\left(c_{0}+c_{1}y\right)}{m}y^{\prime }+\frac{k_{1}y}{m}+\frac{c_{1}c_{0}y^{2}}{3m^{2}}+\frac{c_{1}^{2}y^{3}}{9m^{2}}=0$$

and the Lie condition system (4) becomes as follows.

$$w_{x}-z\;w+\frac{c_{1}}{3m}=0$$

$$w_{y}+w^{2}=0$$

$$9z_{x}\;m^{2}+\left(y^{3}c_{1}^{2}+9yk_{1}m+3y^{2}c_{1}c_{0}\right)w-9z\;m^{2}+9m\left(c_{0}+c_{1}y\right)z-3\;c_{1}^{2}y^{2}\;\;-6\;c_{1}c_{0}y-9k_{1}m=0$$

$$z_{y}+z\;w-\frac{2\;c_{1}}{3\;m}=0$$

This system has a particular solution $w(x,y)=\frac{1}{y}$, $z(x,y)=\frac{c_{1}y}{3m}$. Then the system (6) becomes

$$ys_{yy}+2s_{y}=0$$

$$3\;s_{xy}\;ym+3\;s_{x}m-c_{1}y^{2}s_{y}=0$$

$$s_{xx}+\left(\frac{c_{0}+c_{1}y}{m}-\frac{2c_{1}y}{3m}\right)s_{x}-\left(\frac{k_{1}y}{m}+\frac{c_{0}c_{1}y^{2}}{3m^{2}}+\frac{c_{1}^{2}y^{3}}{9m^{2}}\right)s_{y}=0$$

This system has two solutions with nonzero Jacobian for our considered parameters that provide the point transformations as

$$\psi (x,y)=\frac{e^{-\frac{c_{0}x}{2m}}}{6k_{1}my}\left(c_{1}\sqrt{4k_{1}m-c_{0}^{2}}\;y\cos(\omega )+\left(c_{1}c_{0}y+6k_{1}m\right)\sin(\omega )\right)$$

and

$$\phi (x,y)=\frac{e^{-\frac{c_{0}x}{2m}}}{6k_{1}my}\left(\left(c_{0}c_{1}y+6k_{1}m\right)\cos(\omega )-c_{1}\sqrt{4k_{1}m-c_{0}^{2}}\;y\sin(\omega )\right)$$

where

(8) $$\omega =\frac{\sqrt{4k_{1}m-c_{0}^{2}}}{2m}x_{0}.$$

The nonzero Jacobian

$$J=\frac{e^{-\frac{c_{0}x}{m}}\sqrt{4k_{1}m-c_{0}^{2}}}{2my^{3}}$$

transforms the ODE (7) to the free particle equation $u_{tt}=0$. So, the general solution of ODE (7) can be written as

(9) $$y(x)=\begin{array}{c} -\frac{6k_{1}m\left(s_{1}\cos(\omega )-\sin(\omega )\right)}{\left(s_{1}c_{0}c_{1}-c_{1}\sqrt{4k_{1}m-c_{0}^{2}}\right)\cos(\omega )-\left(c_{0}c_{1}+s_{1}c_{1}\sqrt{4k_{1}m-c_{0}^{2}}\right)\sin(\omega )-6k_{1}s_{2}me^{\frac{c_{0}x}{2m}}} \end{array}$$

where *ω* is defined in (8), $s_{1}$ and $s_{2}$ are arbitrary constants.

One can see that general solution of equation (7) in the linear case when $c_{1}=0$ can be obtained as a constant multiple of that solution given by substituting $c_{1}=0$ in equation (9) after relabeling the constants as follows:

$$y(x)=e^{-\frac{c_{0}x}{2m}}\left(A\cos(\omega ))+B\sin(\omega )\right)$$

Now, we will provide the values of $s_{1}$ and $s_{2}$ for the two following cases:

**Case1:** (Initial displacement and the velocity for some non-zero instant)

For the conditions y(0)=0, $y^{\prime }(x_{0})=0$, $x_{0}\neq 0$, the values of $s_{1}$ and $s_{2}$ can be given as

$$s_{1}=0$$

and

$$s_{2}=\frac{c_{1}e^{-\frac{c_{0}x_{0}}{2m}}\left(c_{0}^{2}-4k_{1}m\right)}{6k_{1}m\left(\sqrt{4k_{1}m-c_{0}^{2}}\;\;\cos(\omega )-c_{0}\sin(\omega ))\right)}$$

Note that in the linear case y=0.

**Case2:** (Initial displacement and the non-zero displacement for some non-zero instant)

For the conditions y(0)=0, $y(x_{0})=y_{0}$, $x_{0}\neq 0,y_{0}\neq 0$, the values of $s_{1}$ and $s_{2}$ can be given as

$$s_{1}=0$$

$$s_{2}=-\frac{e^{-\frac{c_{0}x_{0}}{2m}}}{6y_{0}k_{1}m}\left(6mk_{1}\sin(\omega )+c_{1}y_{0}\sqrt{4k_{1}m-c_{0}^{2}}\cos(\omega )+c_{0}c_{1}y_{0}\;\sin(\omega )\right)$$

Moreover, in the linear case

$$s_{1}=0$$

$$s_{2}=-\frac{e^{-\frac{c_{0}x_{0}}{2m}}}{y_{0}}\sin(\omega )$$

**Case3:** (Initial displacement and initial velocity)

For the conditions $y(0)=y_{0}$, $y^{\prime }(0)=y_{1}$, the values of $s_{1}$ and $s_{2}$ can be given as

$$s_{1}=-\frac{3\sqrt{4k_{1}m-c_{0}^{2}}y_{0}}{6y_{1}m+3c_{0}y_{0}+2c_{1}y_{0}^{2}}$$

$$s_{2}=-\frac{\left(c_{1}^{2}y_{0}^{2}+\left(3y_{1}m+3c_{0}y_{0}\right)c_{1}+9k_{1}m\right)\sqrt{4k_{1}m-c_{0}^{2}}}{3k_{1}m\left(6y_{1}m+3c_{0}y_{0}+2c_{1}y_{0}^{2}\right)}$$

In the linear case:

$$s_{1}=-\frac{\sqrt{4k_{1}m-c_{0}^{2}}y_{0}}{2y_{1}m+c_{0}y_{0}}$$

$$s_{2}=-\frac{\sqrt{4k_{1}m-c_{0}^{2}}}{2y_{1}m+c_{0}y_{0}}$$

A computer code is developed to simulate the non-linear flexural characteristics of the cantilever beam for three cases considered. The physical properties used in the simulations are given in Table 1.

**Table 1:** Properties used in the simulations.

Property	Numerical value
*c*_0_	0.42 Ns/m
*c*_1_	368 Ns/m^2^
*k*_1_	875 N/m
*k*_2_	416.323 N/m^2^
*k*_3_	1.216 × 10^5^ N/m^3^
*m*	0.12375 kg
*y*_0_	0.04 m
*y*_1_	10 m/s
*x*_0_	0.5 s

## 4 Results and discussion

The closed form solution for the non-linear equation of motion governing the cantilever beam displacement is obtained using the Lie-Tresse linearization method through linearizing the governing equation of motion. The analytical study covers three conditions associated with the initial and boundary conditions of the cantilever beam prior to the flexural motion. These conditions include:

• Case 1: initial displacement and the velocity are known for some non-zero instant of time.
• Case 2: initial displacement is non-zero and the displacement is known for some instant of time other than zero.
• Case 3: initial displacement and initial velocity are known.

The linear motion of the cantilever beam is also considered and the analytical solution obtained for three conditions is reduced to a linear solution after introducing the appropriate beam damping and stiffness parameters.

**Figure 2:**
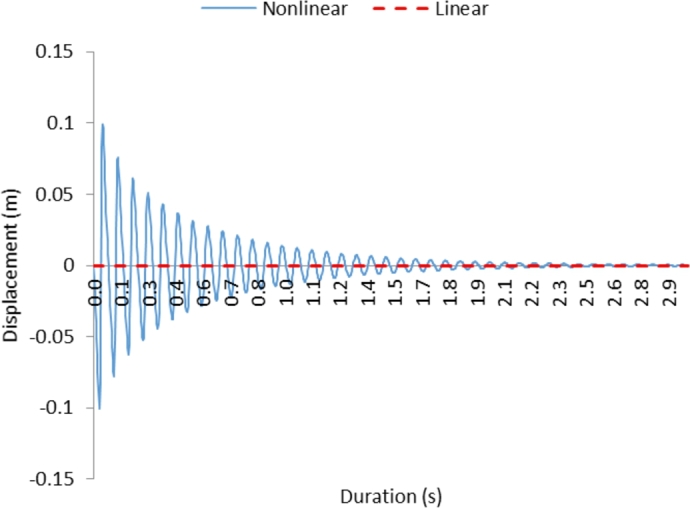
Displacement with time for case 1; for the condition *y*(0)=0, *y*′(*x*_0_)=0, *x*_0_ ≠ 0.

Fig. 2 shows displacement with time curves for non-linear and linear behavior of the cantilever beam for the first case. It should be noted that the conditions for the case 1 are y(0)=0, $y^{\prime }(x_{0})=0$, $x_{0}\neq 0$, which represent the zero initial displacement and non-zero velocity at instant of time other than the initial time. Oscillation of the cantilever beam damps at a faster rate in the early periods and as the period progresses, the rate of damping reduces. This is associated with the damping coefficient and stiffness of the cantilever beam incorporated in the analysis, which are in the form of $c=c_{0}+c_{1}y$, and $k=k_{1}+k_{2}y+k_{3}y^{2}$. Since the value of *c*_1_ is much larger than *c*_*o*_, the damping rate becomes larger during the early periods where the amplitude is large (*y*). Since the linear and the quadratic forms of damping coefficient and stiffness are considered in the analysis, no sudden jump in the amplitude is observed during the oscillation. Despite the behavior of the beam is non-linear, the displacement characteristics appear to be linear in the figure because of the continuous functional relation between the damping coefficient and the displacement. In the case of a linear cantilever beam, the beam does not respond to the conditions introduced in the analysis; in which case, amplitude remains zero for all the periods incorporated in the analysis.

**Figure 3:**
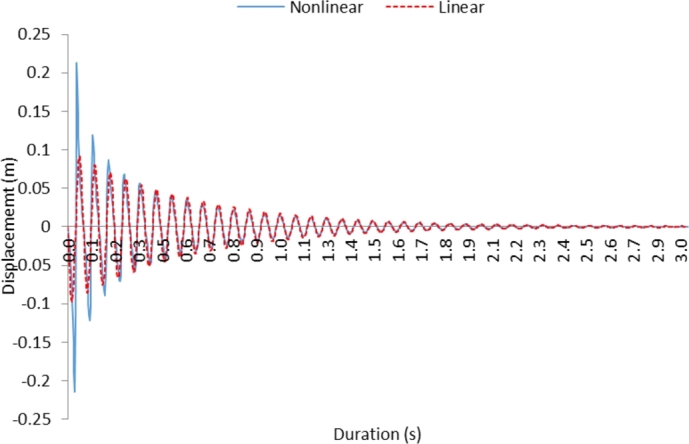
Displacement with time for case 2; for the condition *y*(0)=0, *y*(*x*_0_)=*y*_0_, *x*_0_ ≠ 0,*y*_0_ ≠ 0.

Fig. 3 shows displacement characteristics of the cantilever beam for the case 2; in which case, y(0)=0, $y(x_{0})=y_{0}$, $x_{0}\neq 0,y_{0}\neq 0$ conditions are imposed. These conditions represent that initially the beam is in rest and displacement is zero; at any instant of time, which is different from the initial time, displacement is known (*y*_*o*_) and all times other than the initial time, displacement is not zero. Similar to the previous behavior as shown in Fig. 2, the rate of damping is high during the early periods and amplitude decays gradually with the progressing period. The linear behavior of the beam results in lower amplitude than the non-linear beam; provided that as the time progresses the oscillation becomes almost the same for non-linear and linear characteristics of the cantilever beam. This is attributed to the low values of amplitude (*y*) at long oscillation durations; hence, the effect of $c_{1}y$ on the damping coefficient becomes less and the cantilever beam behaves like a linear beam. Moreover, the effects of non-linear characteristics of the cantilever beam are more pronounced on the amplitude of oscillation as compared to the frequency. This is again because of the linear and quadratic variation of the damping constant and the stiffness of the cantilever beam with the displacement. Therefore, no frequency shift or jump is observed during the oscillation of the non-linear cantilever beam.

**Figure 4:**
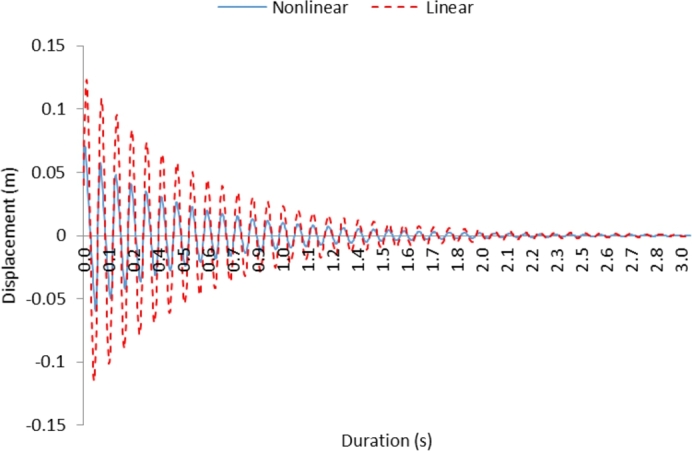
Displacement with time for case 3; for the condition *y*(0)=*y*_0_, *y*′(0)=*y*_1_.

Fig. 4 shows oscillation characteristics of linear and non-linear cantilever beams for the conditions introduced in case 3. The conditions incorporated for the case 3 are $y(0)=y_{0}$, $y^{\prime }(0)=y_{1}$; in which case, initially the displacement is assumed to be non-zero (*y*_*o*_) and the initial velocity ($y^{\prime }(0)$) is also considered to be non-zero. In this case, the amplitude of non-linear behavior of the cantilever beam becomes less than that corresponding to the linear cantilever beam. This is associated with the high values of the damping coefficient during the initial oscillation of the non-linear cantilever beam. Therefore, the linear variation of damping coefficient with the amplitude as well as the parabolic behavior of stiffness acts as constraints on the oscillation of the non-linear cantilever beam. This behavior lowers the decay rate of damping of the oscillation with the progressing period. Therefore, energy dissipation through a non-linear cantilever beam becomes slower than that of the linear beam. Moreover, as similar to those observed for Figure 2, Figure 3, no frequency shift takes place between the linear and non-linear cantilever beam behaviors. The non-linear effect appears to be significant only for the amplitude of the oscillation. This behavior is associated with the linear variation of the damping coefficient of the non-linear cantilever beam.

In general, the linear/nonlinear case responses *y* can be written/approximated as $y=e^{-cx}[A_{1}\sin(\omega_{d}x)+A_{2}\cos(\omega_{d}x)]$, where *A*_1_ and *A*_2_ are initial condition-dependent constants, *c* is damping factor controlling response decay or envelope, and $\omega_{d}$ is frequency of damped response. For the two cases (Cases 2 and 3), it appears that the frequency of damped response $\omega_{d}$ is same for both the linear and nonlinear models. As for the decay of response, the damping factor *c* is greater for the nonlinear model than the linear one, causing the nonlinear response to attenuate with greater rate than the linear response.

## Declarations

### Author contribution statement

B.S. Yilbas and M. Sunar: Analyzed and interpreted the data; Contributed reagents, materials, analysis tools or data; Wrote the paper.

Raed Ali Marabeh; Ahmad Y Al-Dweik: Contributed reagents, materials, analysis tools or data; Wrote the paper.

### Funding statement

The Open Access funding for this article was provided by the Qatar National Library.

### Data availability statement

No data was used for the research described in the article.

### Declaration of interests statement

The authors declare no conflict of interest.

### Additional information

No additional information is available for this paper.
